# Gelatin Nanoparticles for Complexation and Enhanced Cellular Delivery of mRNA

**DOI:** 10.3390/nano12193423

**Published:** 2022-09-29

**Authors:** Lea Andrée, Rik Oude Egberink, Josephine Dodemont, Negar Hassani Besheli, Fang Yang, Roland Brock, Sander C. G. Leeuwenburgh

**Affiliations:** 1 Department of Dentistry—Regenerative Biomaterials, Radboud Institute for Molecular Life Sciences, Radboud University Medical Center, Philips van Leydenlaan 25, 6525 EX Nijmegen, The Netherlands; 2 Department of Biochemistry, Radboud Institute for Molecular Life Sciences, Radboud University Medical Center, Geert Grooteplein 28, 6525 GA Nijmegen, The Netherlands; 3 Department of Medical Biochemistry, College of Medicine and Medical Sciences, Arabian Gulf University, Manama 329, Bahrain

**Keywords:** gelatin, gelatin nanoparticles, mRNA, mRNA delivery, endosomal escape

## Abstract

Messenger RNA (mRNA) is increasingly gaining interest as a modality in vaccination and protein replacement therapy. In regenerative medicine, the mRNA-mediated expression of growth factors has shown promising results. In contrast to protein delivery, successful mRNA delivery requires a vector to induce cellular uptake and subsequent endosomal escape to reach its end destination, the ribosome. Current non-viral vectors such as lipid- or polymer-based nanoparticles have been successfully used to express mRNA-encoded proteins. However, to advance the use of mRNA in regenerative medicine, it is required to assess the compatibility of mRNA with biomaterials that are typically applied in this field. Herein, we investigated the complexation, cellular uptake and maintenance of the integrity of mRNA complexed with gelatin nanoparticles (GNPs). To this end, GNPs with positive, neutral or negative surface charge were synthesized to assess their ability to bind and transport mRNA into cells. Positively charged GNPs exhibited the highest binding affinity and transported substantial amounts of mRNA into pre-osteoblastic cells, as assessed by confocal microscopy using fluorescently labeled mRNA. Furthermore, the GNP-bound mRNA remained stable. However, no expression of mRNA-encoded protein was detected, which is likely related to insufficient endosomal escape and/or mRNA release from the GNPs. Our results indicate that gelatin-based nanomaterials interact with mRNA in a charge-dependent manner and also mediate cellular uptake. These results create the basis for the incorporation of further functionality to yield endosomal release.

## 1. Introduction

Growth factors (GFs) are widely used in tissue engineering and regenerative medicine to stimulate cell differentiation [1]. However, the use of GFs in the clinic has proven difficult due to their short half-life in the range of minutes to hours [2]. To compensate for the fast degradation, supraphysiological doses of growth factors have been clinically administered, which resulted in severe side effects and even malignancies [3,4,5,6].

Alternatively, messenger RNA (mRNA), as the intermediary between gene and protein expression, has recently emerged as a new class of therapeutic agents for the prevention and treatment of various diseases [7]. Since mRNAs are large (300–5000 kDa) and negatively charged macromolecules that do not pass through the lipid bilayer of cell membranes, complexation with a carrier (vector) into a sub-micron nanoparticle is required to enable cellular uptake and delivery into the cytosol [8]. Non-viral vectors such as lipid- or polymer-based nanoparticles are preferred over viral ones due to their superior safety profile. Generally, mRNA-nanoparticles as non-viral vectors are formed through electrostatic interactions between the negatively charged mRNA and cationic (protonatable) lipids or polymers [8,9,10].

Regenerative medicine is a highly attractive area of application for mRNA. The functionalization of biomaterials with therapeutic agents that enable the transient expression of a therapeutic protein over several days can enhance the regenerative potential of these functionalized biomaterials as compared to non-functionalized materials [11]. Several groups have explored the combination of mRNA-nanoparticles and biomaterials or scaffolds to facilitate the local delivery of mRNA [12,13,14,15,16,17]. Recently, De La Vega et al. showed that the local delivery of a chemically modified BMP-2 mRNA enabled the healing of large segmental bone defects in rats without the formation of a massive callus as observed for recombinant human BMP-2 protein [11]. However, all of these studies relied on the use of conventional fibrous collagen biomaterials, which are less suited for application as an injectable biomaterial, and in particular for filling irregularly shaped tissue defects.

Gelatin, as a derivative of collagen, is inherently biocompatible and biodegradable [18]. In the form of gelatin nanoparticles (GNPs), the high surface area and amphoteric (ability to react as an acid or base) nature of gelatin allows for the efficient loading of various biomolecules, including growth factors, antibiotics, and RNA [19,20,21]. By tuning particle crosslinking density—and thus the degradation rate—of GNPs, different release profiles can be obtained [19,22]. Moreover, our group has previously shown that gelatin nanoparticles can be assembled into a network of particle strands and form colloidal hydrogels [23]. Since the particles are reversibly bound within the colloidal networks [24,25], these colloidal gelatin gels fluidize under shear stress and are able to (partially) recover their initial mechanical properties afterwards in a process called self-healing, thereby facilitating minimally invasive administration [23]. GNPs can thus serve as (i) carriers for therapeutic biomolecules and (ii) injectable scaffolds for tissue regeneration. This renders gelatin-based nanomaterials interesting candidates for mRNA-delivering biomaterials. Tabata et al. already confirmed the efficacy of gelatin hydrogels and nanoparticles for short interfering RNA (siRNA) encapsulation [26,27]. However, GNPs have not yet been tested as a potential vehicle for mRNA delivery.

The effect of surface charge on the internalization of nanoparticles is currently debated. Most studies agree that, in serum-free conditions, positively charged NPs are preferentially internalized compared to neutral and negatively charged ones [28,29]. However, for the complexation of mRNA, positive charges would be required as well, which would in part be compensated by mRNA binding [30]. Once exposed to a protein-containing physiological environment, the formation of a protein layer, the so-called protein corona, would further modify this surface charge [31]. Therefore, mRNA binding capacity and cellular internalization may be subject to a difficult-to-predict interplay.

Therefore, we investigated the suitability of differently charged GNPs as non-viral vectors for the delivery of mRNA. To this end, GNPs with a positive, neutral or negative surface charge were synthesized to compare their ability to bind and transport mRNA into cells. More specifically, we (i) loaded mRNA onto GNPs and measured their binding and release kinetics, (ii) assessed the internalization of mRNA-loaded GNPs by pre-osteoblastic cells, and (iii) studied their ability for the intracellular delivery of mRNA with and without the stimulation of endosomal escape.

## 2. Materials and Methods

### 2.1. Synthesis of Gelatin Nanoparticles

Gelatin type A (Bloom number 285) and type B (Bloom number 247) were kindly provided by Rousselot (Ghent, Belgium) for the synthesis of neutral and negatively charged GNPs, respectively. Gelatin nanoparticles (GNPs) were obtained by a two-step desolvation method with acetone, as described in detail elsewhere [23]. The first desolvation step was only carried out for type A gelatin to precipitate the high-molecular-weight gelatin. In brief, 25 g of gelatin type A was dissolved in 500 mL demineralized water under stirring (450 rpm) at 45 °C. Subsequently, the stirring speed was increased to 1000 rpm, and 500 mL acetone was rapidly added. After 3 min, the suspension was left to cool down without stirring for 15 min. Thereafter, the supernatant was discarded, and the remaining high-molecular-weight gelatin was dissolved in 450 mL demineralized water and freeze-dried for 48 h. In the second desolvation step, 2.5 g gelatin was dissolved in 50 mL demineralized water under stirring (400 rpm) at 45 °C. The pH was adjusted to 2.5 using 6 M HCl (37% fuming, Merck Millipore, Burlington, MA, USA), after which 134 mL (type A) and 138 mL (type B) acetone (Boom, Meppel, The Netherlands) were added dropwise (8 mL/min) under stirring (1000 rpm) to induce gelatin desolvation into spherical nanoparticles. Subsequently, 316 μL glutaraldehyde (25 wt% aqueous solution, Acros Organics, Geel, Belgium) was added and left for 16 h under stirring (400 rpm) at room temperature (RT) to crosslink the nanoparticles. The reaction was stopped by adding 100 mL of 100 mM glycine (Sigma-Aldrich, St. Louis, MO, USA) solution. The GNPs were collected by centrifugation (24,000 rcf, 40 min) and washed two times by redispersion in demineralized water. To synthesize positively charged nanoparticles, washed GNPs prepared from gelatin A as described above were dispersed in 80 mL of phosphate-buffered saline (without calcium, magnesium and sodium pyruvate, sterile-filtered, Gibco, Carlsbad, CA, USA) at pH 5.5, whereupon carboxyl groups of the gelatin nanoparticles were activated with 383 mg of 1-Ethyl-3-(3-dimethylaminopropyl)carbodiimide (EDC, Sigma-Aldrich) by stirring at 300 rpm for 20 min at RT. Subsequently, surface amination was carried out by the addition of 1.708 mL of ethylenediamine (VWR Chemicals, Radnor, PA, USA). The pH was re-adjusted to 5.5, and the suspension was left to react overnight. The next day, nanoparticles were washed trice with demineralized water (16,800 rcf, 20 min). The washed GNPs were dispersed in 60 mL demineralized water and kept at 4 °C until further use.

### 2.2. Gelatin Nanoparticle Characterization

#### 2.2.1. Morphology, Size, Zeta Potential

The hydrodynamic diameter of GNPs was determined in demineralized water by dynamic light scattering using a Malvern Zetasizer Nano-Z (Malvern Instruments, Worcestershire, UK), while the zeta-potential of GNPs was measured in 5 mM HEPES buffer (Sigma-Aldrich) at pH 7.4. To visualize their morphology, GNPs were freeze-dried in an acetone/water mixture (30/70 *v*/*v*%), sputter-coated with 10 nm chromium, and imaged using a Zeiss Sigma 300 field-emission scanning electron microscope (SEM). The average size in a dry state was determined by measuring the diameter of 100 nanoparticles in SEM images using open-source Fiji software.

#### 2.2.2. Gelatin Nanoparticle Degradation

The degradation of GNPs was assessed in the presence and absence of collagenase, an enzyme capable of digesting gelatin. A total of 1 mg of GNPs was weighed and rehydrated overnight in 500 μL demineralized water. The next day, swollen particles were collected by centrifugation (15,000 rcf, 10 min), resuspended in 500 μL of degradation solution (0.4 mM CaCl_2_ in PBS (pH 7.4) with or without the addition of 400 ng/mL collagenase 1A (Sigma-Aldrich)), and subsequently incubated on a shaking plate at 37 °C. At the respective time points, the supernatant was collected by centrifugation (15,000 rcf, 10 min), and particles were resuspended in a freshly prepared degradation medium. The total protein content in the supernatant was determined using the Micro BCA protein assay kit (ThermoFisher Scientific, Waltham, MA, USA) according to the manufacturer’s instruction but using a standard curve prepared with gelatin type A or type B accordingly.

### 2.3. Loading of GNPs with mRNA and mRNA Release Kinetics

Uncapped secreted nanoluciferase (SecNLuc) mRNA (RiboPro, Oss, The Netherlands) was used as a model mRNA to determine mRNA binding efficiency and retention. The experiments were performed in RNase-free demineralized water to avoid charge changes of the nanoparticles due to ions present in physiological buffers. 10 μg GNPs were collected by centrifugation and resuspended in 50 μL RNase-free demineralized water containing 200 ng of SecNLuc mRNA. The suspension was mixed thoroughly and incubated overnight at 4 °C. The next day, the particles were collected by centrifugation, and the unbound mRNA in the supernatant was quantified by means of the QuantiFluor RNA assay (Promega, Madison, WI, USA). In brief, 10 μL supernatant or standard were mixed with 50 μL dye solution (QuantiFluor dye 1:2000 in Tris-EDTA (TE) buffer) in a 384-well plate and incubated for 5 min at RT protected from light before measuring the fluorescence at 540 nm upon excitation at 492 nm. The standard curve was prepared by a twofold dilution series of the original SecNLuc mRNA stock.

To assess the binding strength of loaded SecNLuc, a desorption assay with the polyanion heparin (heparin sodium salt from porcine intestinal mucosa, Sigma-Aldrich) was performed. Of note, heparin was used in large excess to maximize the desorption of mRNA through competitive binding. After SecNLuc loading on GNPs, the particles were collected and resuspended in 200 µL RNase-free TE buffer with or without 320 µg heparin. After incubation for 60 min at 37 °C, the particles were collected by centrifugation (15,000 rcf, 5 min), and the supernatants were analyzed using the QuantiFluor RNA assay. Furthermore, the dissociation of mRNA-GNP complexes was monitored as a function of time to investigate the mRNA release kinetics. To this end, SecNLuc was loaded onto GNPs as described above and washed once with demineralized water to remove loosely bound mRNA. Then, 50 μL of RNase-free Milli-Q water was added, and the mRNA-loaded GNPs were incubated under static conditions at 37 °C. At the respective time points, the mRNA-loaded GNPs were spun down (15,000 rcf, 10 min), the supernatant was collected, and fresh demineralized water was added. Supernatants were stored at −20 °C until analysis using the QuantiFluor RNA assay.

### 2.4. RNA Integrity Assay

The RNA standard (1.2 kb) of the QuantiFluor RNA assay was used as model RNA to investigate RNA integrity after loading on GNPs and potential protection of RNA from degradation by RNAse. 40 μg GNPs were collected by centrifugation and resuspended in 8 μL RNase-free demineralized water containing 800 ng of standard RNA. The suspension was mixed thoroughly and incubated overnight at 4 °C. The next day, the particles were collected by centrifugation and washed once with 20 μL RNase-free demineralized water to remove loosely bound RNA. To test protection against RNase, RNA-loaded GNPs were incubated in 8 μL RNase-free demineralized water without or with additional 2 μL of RNase A (0.03 U/mL, Invitrogen, Carlsbad, CA, USA) for 30 min and 2 h, respectively. Thereafter, the RNase was removed by centrifugation and GNPs were washed once with 20 μL RNase-free demineralized water. Prior to gel electrophoresis, the RNA was desorbed from GNPs by incubation with 7 μL heparin in 3 μL RNase-free demineralized water for 45 min at 37 °C. RNA incubated for 30 min with either 10 μL demineralized water or 2 μL RNase A and 8 μL RNase-free demineralized water was used as GNP-free controls. RNA integrity was analyzed by agarose gel electrophoresis. A 1 *w*/*v*% agarose gel was prepared in 0.5x Tris-Borate-EDTA (TBE) buffer. After the dissolution of the agarose, 0.01 *v*/*v*% SYBR Safe DNA stain (Invitrogen) and 1 *v/v*% common household bleach (<5% sodium hypochloride, Piek) were added to visualize RNA and to inactivate RNases present in the gel, respectively [32]. Gels were placed in the electrophoresis apparatus and submerged with 0.5x TBE buffer. 10 μL of sample were loaded, and the gels were run for 50 min at 60 V prior to imaging under UV light (GelDoc EZ Imager, BioRad, Veenendaal, The Netherlands).

### 2.5. Cell Culture

The murine pre-osteoblast cell line MC3T3-E1 subclone 4 (CRL-2593, American Type Culture Collection, Manassas, VA, USA) was maintained at sub-confluency in Minimal Essential Medium α (Gibco, MEM-α without ascorbic acid), supplemented with 10% FBS and 100 units/mL penicillin and 0.1 mg/mL streptomycin (Sigma-Aldrich). These cells were selected since the ultimate aim of this study was to stimulate bone regeneration by transfection with BMP-2 mRNA.

### 2.6. Internalization of Gelatin Nanoparticles

#### 2.6.1. Fluorescent Labeling of GNPs

To allow for the visualization of cellular GNP uptake, GNPs were fluorescently labeled with Alexa Fluor 488 succinimidyl ester (Invitrogen) using NHS-amine coupling. 10 mg GNPs were dispersed in 1 mL PBS at pH 7.4, followed by the addition of 2.5 μg of fluorophore-ester in 10 μL dimethylformamide (Serva, Tulsa, OK, USA). The suspension was left to react for 1 h at RT under stirring at 400 rpm. Thereafter, the particles were collected by centrifugation (9700 rcf, 10 min) and washed twice with demineralized water. The particles were stored at a concentration of 1 mg/mL in PBS at 4 °C.

#### 2.6.2. Visualization of Cellular Uptake of Bare and mRNA-Loaded GNPs

10,000 cells/cm^2^ were seeded in an 8-well µ-slide (ibidi, Gräfelfing, Germany) and left to adhere overnight. The next day, cells were stained with 1 µM CellTrace yellow (Invitrogen) in PBS according to the manufacturer’s instructions. Then, 10 μg of either bare Alexa Fluor 488-labeled GNPs or Alexa Fluor 488-labeled GNPs loaded with 200 ng of Alexa Fluor 647-labeled uncapped SecNLuc mRNA (RiboPro) as described previously were diluted in full cell culture medium to reach a final concentration of 50 µg/mL, and 200 μL (10 μg GNPs) was added to the cells. 200 ng of naked (i.e., without GNPs) fluorescently labeled SecNLuc mRNA were added per well as a control group. After 24 h, the GNP-containing medium was removed, and cells were washed twice with PBS and phenol-red-free Roswell Park Memorial Institute medium (RPMI, Gibco) supplemented with 10% FBS, and 20 mM HEPES (Sigma-Aldrich) was added for image acquisition. For samples only containing bare GNPs, lysosomal compartments were stained with 50 nM LysoTracker deep red (ThermoFisher Scientific) in phenol-red-free RPMI medium supplemented with 10% FBS and 20 mM HEPES 30 min prior to imaging. The internalization of GNPs was visualized using a Leica TCS SP8 SMD confocal microscope (Leica Microsystems), equipped with an HCX PL APO 63x/0.40 water immersion objective and a temperature-controlled stage at 36.5 °C. Fluorophores were excited using a white-light laser, and emissions were detected with hybrid detectors (HyD). Alexa Fluor 488-labeled GNPs were excited at 488 nm (detection: 500–540 nm); CellTrace yellow was excited at 561 nm (detection: 580–620 nm), and LysoTracker deep red or Alexa Fluor 647-labeled mRNA was excited at 633 nm (detection: 660–700 nm and 655–700 nm, respectively). Fiji was used for the reconstruction and quantification of images.

### 2.7. Expression of mRNA-Encoded Proteins

Cells were seeded and transfected with 10 µg of GNPs loaded with 200 ng of capped, polyadenylated CleanCap EGFP mRNA coding for enhanced green fluorescent protein (eGFP) (mRNA L-7601, 996 nt, TriLink Biotechnologies, San Diego, CA, USA) as described above. After 24 h, the medium containing mRNA-loaded GNPs was removed; cells were washed twice with PBS, and phenol red-free RPMI medium supplemented with 10% FBS and 20 mM HEPES was added for imaging. eGFP was excited at 488 nm (detection: 500–540 nm), and CellTrace yellow was excited at 561 nm (detection: 580–620 nm).

### 2.8. Endosomal Escape Assay

In addition to EGFP expression, the expression of mRNA-encoded protein was also tested using SecNLuc mRNA. 100 µg of GNPs were collected by centrifugation (20,000 rcf, 10 min), and, after the removal of the supernatant, the pellet was resuspended with 2.14 µg of SecNLuc mRNA in 21.46 µL demineralized water, followed by overnight incubation at 4 °C in Protein LoBind tubes (Eppendorf, Hamburg, Germany). After additional centrifugation, the pellet of mRNA-loaded GNPs was resuspended in 200 µL alpha-MEM and added to cells seeded 24 h earlier at a density of 10,000 cells in a tissue-culture-treated 96-well plate (Greiner Bio-One, Kremsmünster, Austria). As a positive control, SecNLuc mRNA complexed with Lipofectamine MessengerMAX (LMM; ThermoFisher Scientific) was used as per the manufacturer’s instructions. In short, LMM was incubated in a volume of Opti-MEM (Gibco) for 10 min at RT. The appropriate mRNA solution was diluted in Opti-MEM and incubated with LMM for at least 5 min at RT. After 2 h of incubation with either GNP-mRNA or LMM-mRNA complexes, 50 µM chloroquine (chloroquine diphosphate salt, Sigma-Aldrich), known to stimulate the disruption of the lysosomal membrane [33], was added either 2, 4 or 24 h post-transfection. Luciferase expression was measured 30 and 50 h post-transfection.

The extent of luciferase expression was determined using the Nano-Glo Luciferase Assay System (Promega) according to the manufacturer’s instructions. Briefly, 50 µL of the sample was mixed with a 1:50 dilution of Nano-Glo luciferase assay substrate in a Nano-Glo luciferase assay buffer. The resulting mixture was incubated at RT and protected from light for at least 3 min in a black clear flat bottom 96-well plate (Corning, New York, NY, USA). Importantly, an inter-sample distance in the 96-well plate of at least two columns prevented crosstalk of signals between different experimental conditions. Luminescence was measured after briefly shaking the plate using the VICTOR X3 Multilabel Plate Reader (Perkin Elmer, Waltham, MA, USA). Untransfected cells were used to determine the background signal.

### 2.9. Statistical Analysis

Statistical analysis was performed with Prism version 8.4 (GraphPad, San Diego, CA, USA). Data were tested for normality with a Shapiro–Wilk test and analyzed by one- or two-way analysis of variance (ANOVA) with Tukey multiple comparison correction to detect differences between the three GNP groups. If requirements for ANOVA were not met, Kruskal–Wallis testing with Dunn multiple comparison correction was used. To compare mRNA desorption with and without heparin for the same GNP type, a *t*-test was used. Experiments were performed in triplicate or quadruplicate, except for the degradation of neutral GNPs, which was performed in duplicate due to the limited availability of nanoparticles. The number (*n*) of samples or analyzed frames are indicated in the respective figure legend. All data are presented as mean ± standard deviation. Significance was set at *p* < 0.05, and *p* values are reported using * *p* < 0.05, ** *p* < 0.01, *** *p* < 0.001 and **** *p* < 0.0001.

## 3. Results and Discussion

### 3.1. Gelatin Nanoparticle Characterization

**Figure 1:**
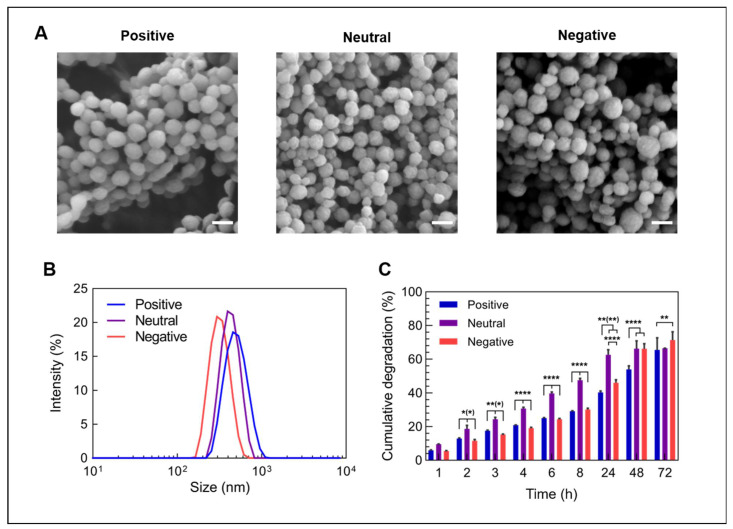
Characteristics of differently charged, bare gelatin nanoparticles. (**A**) SEM images of lyophilized gelatin nanoparticles with different surface charges, (**B**) size distribution of gelatin nanoparticles in an aqueous solution and (**C**) the degradation of gelatin nanoparticles in the presence of collagenase type 1A at pH 7.4 (*n* = 3 for positive and negative particles, *n* = 2 for neutral particles). Scale bars represents 200 nm. Statistical significance is shown as * *p* < 0.05, ** *p* < 0.01, and **** *p* < 0.0001.

The synthesized gelatin nanoparticles (GNPs) were characterized regarding their morphology, (hydrodynamic) size and surface charge, as summarized in Table 1. GNPs made from gelatin type A and type B showed a spherical morphology (Figure 1A) and had a net surface charge of +2.9 mV and −11.6 mV, respectively, in an aqueous suspension buffered at pH 7.4. GNPs had a hydrodynamic wet size of 412 nm (type A) and 306 nm (type B) with a narrow size distribution (polydispersity index (PDI) = 0.028 and 0.046, respectively), as shown in Figure 1B. The surface modification of type A GNPs with ethylenediamine increased the zeta potential to +19 mV without compromising their spherical morphology. After this modification, the hydrodynamic size increased to 471 nm (PDI = 0.079). Dynamic light scattering (DLS) measures particle size based on the particle diffusion rate. As one possible explanation, an increased surface charge can lead to an extended ion layer around the particle core, resulting in a decreased diffusion rate and, thus, an apparent larger hydrodynamic diameter [34]. Alternatively, after the introduction of positive charges, the gelatin could be less condensed. However, all particle types showed a similar size in a dry state as determined from SEM images, confirming that this surface modification did not change the morphology or dry particle size (Table 1).

When soaked in a collagenase-containing solution at 37 °C, all three particle types degraded rapidly within several days (Figure 1C). Neutral GNPs degraded most rapidly, as evidenced by 50% degradation after approximately 10 h, while positively and negatively charged GNPs reached 50% degradation after about 48 h and 30 h, respectively. Nevertheless, all GNP types showed continuous degradation reaching approximately 70% after 72 h. In contrast, the GNPs did not degrade (<1%) in the absence of collagenase or in lysosomal pH of 5.5. These data are in accordance with previously published work and confirm the enzymatic degradation pathway of the GNPs [20].

Upon loading of mRNA to the GNPs, all three types of GNPs became positively charged (26.4 mV ± 0.6 mV for positively, 8.3 mV ± 0.5 mV for neutral and 5.0 ± 0.3 mV for negatively charged nanoparticles), which might suggest that mRNA loading resulted in a higher net amount of amine groups exposed at the outer surface of the nanoparticles caused by the positively charged mRNA base pairs.

### 3.2. Loading of GNPs with mRNA and mRNA Release Kinetics

**Figure 2:**
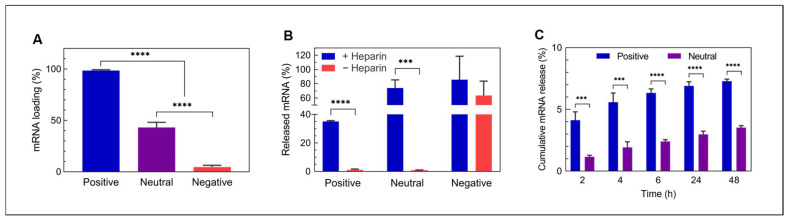
mRNA loading and release from gelatin nanoparticles. (**A**) Loading efficiency for differently charged gelatin nanoparticles, calculated based on the mRNA remaining in the supernatant (*n* = 6), (**B**) competitive mRNA decomplexation from gelatin nanoparticles with heparin (*n* = 3) and (**C**) cumulative spontaneous mRNA release in aqueous suspension (*n* = 3). Release data for negatively charged nanoparticles was not included, due to mRNA amounts below the detection limit. Statistical significance is shown as *** *p* < 0.001 and **** *p* < 0.0001.

A prerequisite of effective mRNA carriers is their ability to bind the negatively charged mRNA. As expected, positively charged GNPs showed a much higher affinity for negatively charged mRNA, with a loading efficiency (LE) of 98.6 ± 0.8%, as compared to GNPs with neutral (LE = 43.2 ± 5.0%) or negative surface charge (LE = 4.7 ± 1.6%), as shown in Figure 2A. In fact, the affinity of mRNA to positively charged GNPs was so high that only 35.0 ± 0.6% of bound mRNA was released in a desorption assay using heparin as a negatively charged competitor macromolecule (Figure 2B). In contrast, 74.0 ± 11.4% and 85.7 ± 32% of bound mRNA were retrieved from neutral and negatively charged GNPs, respectively, indicating that mRNA was bound less strongly to these GNPs. As mRNA needs to become available to the ribosome after internalization into the cell, we assessed the cumulative mRNA release kinetics as a function of time up to 48 h (Figure 2C). Both neutral and positively charged GNPs released mRNA in a continuous manner, reaching 3.5 ± 0.1% and 7.3 ± 0.1% release after 48 h, respectively, whereas the release of mRNA from negatively charged GNPs could not be detected due to little loading in the first place. However, the absolute amounts of released mRNA from neutral GNPs were approximately 5.8-times lower than those from the positively charged ones, also due to the reduced loading efficiency (Figure S1). The limited release of mRNA observed here has also been described by Moràn et al., who found very restricted RNA release from gelatin nanoparticles prepared with protamine sulfate [21]. Other studies investigated the release of biomolecules from gelatin micro- or nanoparticles in the presence of collagenase [27,35]. Under these conditions, biomolecule release is correlated with gelatin degradation.

### 3.3. RNA Integrity Assay

**Figure 3:**
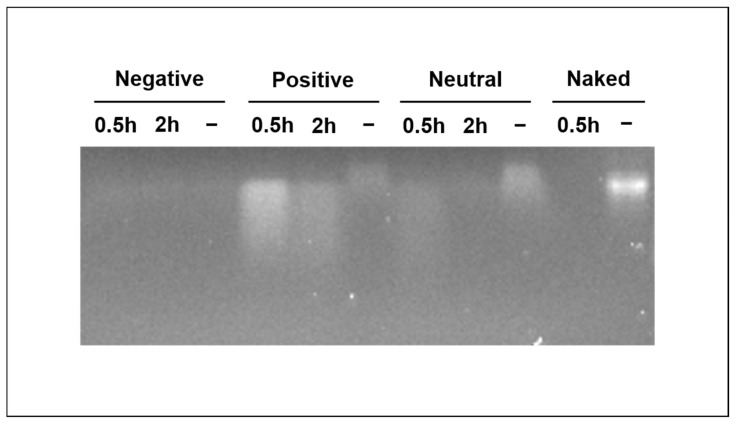
RNA integrity on gelatin nanoparticles. Integrity of RNA loaded to gelatin nanoparticles (lane 1–9) or naked RNA (lane 10 and 11) with (0.5 h and 2 h) or without (−) exposure to RNase A. Naked refers to RNA without any vector or particle.

Messenger RNA is prone to degradation by RNases even after complexation with cationic polymers [36]. For effective mRNA delivery, vectors should thus protect the mRNA from premature degradation. Therefore, we assessed the integrity of RNA loaded on GNPs by gel electrophoresis with and without the exposure to RNase (Figure 3). For positively and neutral charged GNPs, bands of RNA were visible with or without the exposure to RNase, while band intensity was minimal for negatively charged GNPs with or without RNase. Analyzing the loading supernatant clearly showed that RNA was again hardly bound to negatively charged GNPs (Figure S2), confirming the very low loading efficiency on this particle type. For positive and neutral GNPs, the bands of RNase-treated GNPs were weaker than those of the RNase-free conditions, indicating that GNPs do not fully protect RNA from degradation. Moreover, prolonged RNase exposure of 2 h led to weaker bands compared to exposure of 30 min. Generally, positively charged, RNase-treated GNPs showed more intense bands compared to neutral ones, likely due to the stronger interaction of RNA with this particle type. When exposing RNA without a vector (naked) to RNase for 30 min as a control experiment, no signal was detected, indicating the complete degradation of the RNA. As compared to this negative control, the gel electrophoresis results obtained for GNPs suggest that these nanoparticles offer the partial protection of RNA from degradation compared to naked RNA.

### 3.4. Cellular Uptake of GNPs

**Figure 4:**
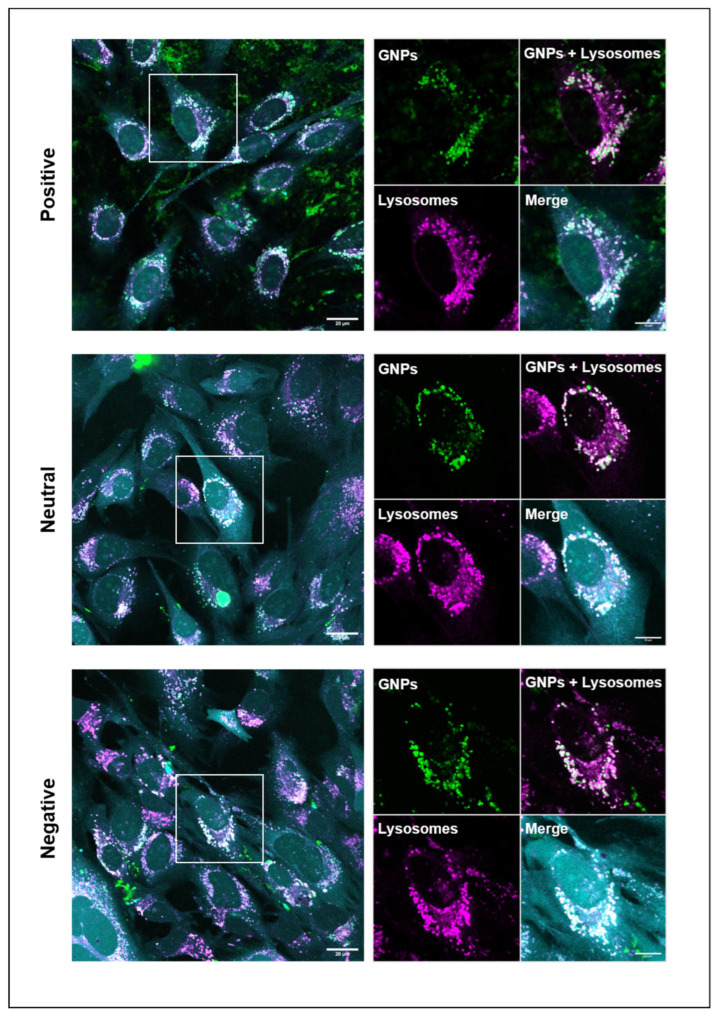
Internalization of gelatin nanoparticles. Confocal live cell images showing internalization after 24 h of differently charged gelatin nanoparticles (green) in mouse pre-osteoblastic cells (cyan) and their localization in lysosomal compartments (magenta) after adjusting image acquisition settings to optimize the visibility of each type of GNPs. The co-localization of GNPs and lysosomal compartments appears white. Enlarged region of interest is marked by white squares in the images on the left. Scale bars represent 20 µm and 10 µm (zoom-in image).

**Figure 5:**
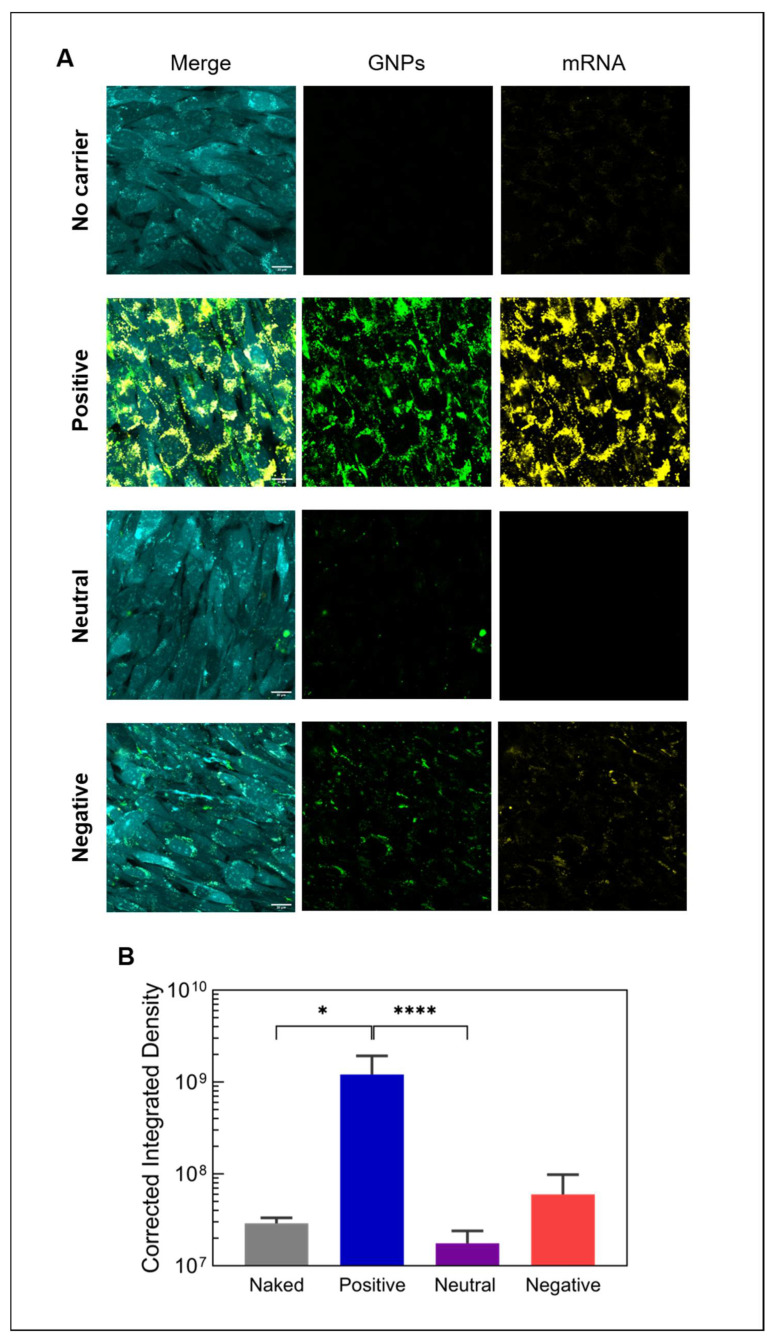
Internalization of mRNA-loaded gelatin nanoparticles. (**A**) Confocal live cell images showing the uptake of fluorescently labeled mRNA (yellow) by mouse pre-osteoblastic cells (cyan) after 24 h using differently charged gelatin nanoparticles (green) and (**B**) the quantification of internalized mRNA (*n* = 6). Naked mRNA refers to mRNA delivered without any vector or particle (no carrier). All images were acquired using the same acquisition settings. Scale bar represents 20 µm. Statistical significance is shown as * *p* < 0.05 and **** *p* < 0.0001.

Any carrier for intracellular (drug) delivery should be sufficiently internalized to enable the effective delivery of its cargo. Therefore, we investigated the cellular uptake of the differently charged GNPs. After 24 h, for all three types of particles, abundant internalization was observed, with a predominant localization in lysosomal compartments (Figure 4), while only limited internalization was observed at earlier time points of 2 and 6 h. For positively charged GNPs, many extracellular nanoparticles were observed even after washing, which was attributed to the strong adsorption to the negative charge of the plasma-treated polystyrene tissue culture plastic. When recording images with the same acquisition parameters for all particle types, fluorescence was higher for positively charged GNPs than for neutral and negatively charged ones. Nevertheless, it should be emphasized that the labeling efficiency of positively charged GNPs was about twofold higher than the one for neutral or negatively charged GNPs (Figure S3), which was caused by the higher amount of amine groups present at the surface of positively charged GNPs. We therefore refrained from the quantification of these images for GNP uptake. Instead, we quantified GNP uptake on images acquired with identical acquisition settings (shown in Figure 5) and corrected for differences in initial fluorescence. No difference in GNP uptake was found between the differently charged GNPs (Figure S4). It should be emphasized that it is increasingly recognized that the effect of nanoparticle charge on cellular uptake is not as simple as often suggested in literature. In contrast to the general assumption that positively charged particles are internalized more efficiently than negative ones, negatively charged particles have also been reported to be internalized more efficiently than positively charged ones [37,38,39], which may be a function of protein corona formation [37].

Since all GNPs were successfully internalized, we tested the ability of all three types of GNPs to deliver mRNA into the cytosol to allow for the expression of mRNA-encoded proteins. The transport of mRNA into cells was quantified using fluorescently labeled mRNA loaded on GNPs. As expected, based on the difference in binding affinity and cellular uptake, positively charged GNPs led to the higher uptake of mRNA (Figure 5A). This observation was confirmed by the quantification of the mRNA signal in the images (Figure 5B), revealing that positively charged GNPs transported 20 times and 69 times more mRNA into cells compared to negatively charged and neutral ones, respectively. Even without a vector (naked mRNA), some mRNA was internalized by cells. Previously, it was reported that many cell types are capable of internalizing naked mRNA via caveolae-/lipid-rafts-dependent routes [40]. In fact, the levels of internalized naked mRNA observed here were not significantly different from the uptake of mRNA delivered with neutral or negatively charged GNPs, indicating that only positively charged GNPs efficiently transport mRNA into cells (Figure 5B).

### 3.5. Expression of mRNA-Encoded Proteins

**Figure 6:**
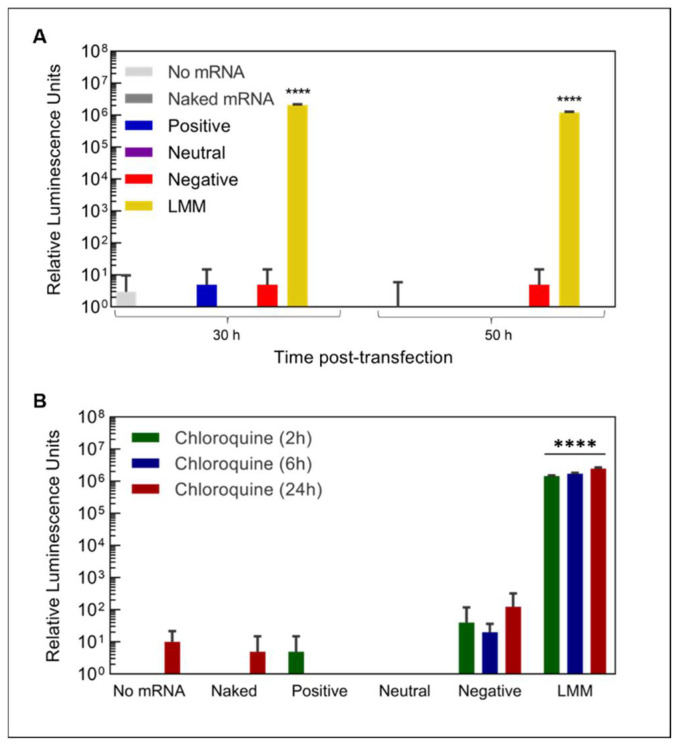
mRNA expression and endosomal release. Expression of luciferase protein (**A**) 30 h and 50 h after transfection with luciferase mRNA loaded on differently charged gelatin nanoparticles or commercial lipoplexes (LMM) and (**B**) 30 h after transfection with the stimulation of endosomal escape by the addition of chloroquine at different time points (*n* = 4). **** indicates statistically significant differences compared to the untransfected control (no mRNA) with *p* < 0.0001.

After showing the successful transport of mRNA into cells, we next assessed the expression of the mRNA-encoded protein using eGFP-encoding mRNA delivered by GNPs using confocal live-cell imaging. Despite the effective mRNA transport by positively charged GNPs into cells, no eGFP expression was observed for any of the particle types. We also tested transfection with a luciferase mRNA, which is known to produce a more sensitive readout [41]. Again, none of the GNPs showed significant protein expression above the experimentally determined background (Figure 6A). In contrast, upon transfection with lipofectamine (LMM), a commercially available transfection agent, protein expression ~10,000 times above background was measured. Of note, although neutral and especially positively charged GNPs showed a high binding affinity for mRNA and continuous release (Figure 2C), only 3.0 ± 0.1% and 6.9 ± 0.2%, respectively, of loaded mRNA had been released within 24 h, the time point of the readout of protein expression. Thus, the lack of detectable protein expression might be caused by the low amounts of available mRNA for translation.

After internalization, endosomal escape is a crucial step in mRNA delivery, wherein mRNA is released from the endo/lysosome and becomes available to the ribosome for translation [33]. We therefore suspected that GNPs lacked the ability to stimulate endosomal escape, since GNPs strongly co-localized with lysosomal structures after 24 h (Figure 4). Nevertheless, even after the addition of the endosomal release agent chloroquine (CQ), no protein expression could be detected for GNP-based mRNA delivery either compared to background or compared to conditions without CQ addition, independent of the GNP type and incubation time with chloroquine (Figure 6B and Figure S5). In contrast, transfection with lipofectamine led to abundant protein expression, regardless of chloroquine addition. These results indicate that GNPs show an insufficient release of mRNA to yield detectable protein expression.

A previous study used GNPs made of cationic gelatin to deliver siRNA, leading to the suppression of target gene expression [27]. Unlike mRNA, siRNAs are short, double-stranded RNAs [42] and generally considered to be more stable. Moreover, siRNA was incorporated during GNP synthesis, which may have increased the resistance against degradation by RNases as well as mRNA loading capacity. Unfortunately, endosomal release was not addressed in the study, leaving the question of how GNPs and/or siRNA evaded the endosome unanswered. The mechanism by which vectors facilitate endosomal escape is still being debated, and most likely depends on the vector as well as the cell type. The proton sponge effect proposes that the (ionizable) vector gets protonated in the acidic environment of the lysosome, resulting in a pH increase within the lysosome. To restore the acidic pH, more protons are pumped in with a subsequent influx of chloride, leading to increased osmotic pressure and ultimately the rupture of the endosome [33,43]. Alternatively, endosomal escape can also be achieved by local destabilization and an increase in the permeability of the membrane through charge-driven interactions between the vector and the endosomal membrane [33,43]. Gelatin contains both cationic and anionic groups along its polymeric backbone [44]. Theoretically, GNPs could stimulate endosomal escape through the proton sponge effect, especially GNPs based on type B gelatin (negatively charged GNPs), which has an isoelectric point around five. However, whether GNPs can indeed increase the lysosomal pH sufficiently to cause an influx of protons is unknown. As far as membrane destabilization is concerned, GNPs do not rapidly degrade in acidic pH, and it seems unlikely that the highly hydrated GNPs can insert into the lipid bilayer of the membrane. Regardless of the possible escape mechanisms, the observations presented herein indicate that GNPs do not lead to the rupture of endosomes or only to a minimal extent. Furthermore, the failure of chloroquine to increase protein expression also suggests that the release of mRNA from the GNP nanoparticles may be compromised. These results highlight the importance of a balance between extracellular robustness and intracellular release/endosomal escape [45,46,47].

## 4. Conclusions and Outlook

mRNA has emerged as a promising new class of therapeutics to enhance the functionality of biomaterials through the expression of regeneration-promoting proteins. We herein investigated GNPs as a potential carrier for mRNA and demonstrated that GNPs enable mRNA complexation and cellular delivery in a charge-dependent manner. Importantly, the complexation of mRNA conferred a protective effect against RNases. Specifically, positively charged GNPs exhibited the highest binding affinity and transported substantial amounts of mRNA into pre-osteoblastic cells. However, no expression of mRNA-encoded protein was detected, which is likely related to the insufficient endosomal escape and/or mRNA-release from the GNPs. Our results thus create a strong basis for further studies to achieve pH-dependent mRNA release and incorporate functionalities that mediate endosomal release.

## Figures and Tables

**Table 1:** Characteristics of bare gelatin nanoparticles.

	Positive	Neutral	Negative
**Gelatin type**	modified A	A	B
**Zeta potential (mV)**	19.0 ± 0.8	2.9 ± 0.3	−11.6 ± 0.8
**Size _hydro_ (nm)**	471 ± 9	412 ± 7	306 ± 8
**Size _dry_ (nm)**	128 ± 18	104 ± 23	133 ± 43

## Data Availability

The data presented in this study are available on request from the corresponding author.

## Supplementary Materials

The following supporting information can be downloaded at: https://www.mdpi.com/article/10.3390/nano12193423/s1, Figure S1: mRNA release from gelatin nanoparticles. Absolute amounts of mRNA released from gelatin nanoparticles over 48 h (*n* = 3). Data for negatively charged nanoparticles were not included since detected mRNA amounts were below the detection limit. Statistical significance is shown as *** *p* < 0.001 and **** *p* < 0.0001. Figure S2: Loading capacity of gelatin nanoparticle. Gel electrophoresis of RNA remaining in supernatant after loading gelatin nanoparticles with RNA. Figure S3: Fluorescent labelling efficiency of gelatin nanoparticles. Fluorescence intensity of equal amounts of fluorescently-labelled gelatin nanoparticles (*n* = 3). Figure S4: Internalization of gelatin nanoparticles. Quantification of internalization of gelatin nanoparticles by pre-osteoblastic cells after 24 h after normalization of original fluorescence based on Figure S3 (*n* = 6). ns indicates no statistically significant differences. Figure S5: Endosomal release of mRNA. Expression of luciferase mRNA 50 h after transfection with differently charged gelatin nanoparticles or commercial lipoplexes (LMM) and stimulation of endosomal escape by addition of chloroquine at different time points (*n* = 4). **** indicates statistically significant differences compared to untransfected control (no mRNA) with *p* < 0.0001.
