# Supplementary material for: Gelatin Nanoparticles for Complexation and Enhanced Cellular Delivery of mRNA
Source: Nanomaterials (Basel). 2022 Sep 29;12(19):3423. doi: 10.3390/nano12193423 (PMC9565693; doi:10.3390/nano12193423)
Supplement: Supplementary file 1 [file nanomaterials-12-03423-s001.zip › nanomaterials-1918675-supplementary-done.pdf]

nanomaterials

Supplementary Materials

Gelatin Nanoparticles for Complexation and Enhanced Cellular Delivery of mRNA

Lea Andrée [1,†], Rik Oude Egberink [2,†], Josephine Dodemont [1], Negar Hassani Besheli [1], Fang Yang [1], Roland Brock [2,3] and Sander C. G. Leeuwenburgh [1,*]

[1] Department of Dentistry—Regenerative Biomaterials, Radboud Institute for Molecular Life Sciences, Radboud University Medical Center, Philips van Leydenlaan 25, 6525 EX Nijmegen, The Netherlands
[2] Department of Biochemistry, Radboud Institute for Molecular Life Sciences, Radboud University Medical Center, Geert Grooteplein 28, 6525 GA Nijmegen, The Netherlands
[3] Department of Medical Biochemistry, College of Medicine and Medical Sciences, Arabian Gulf University, Manama 329, Bahrain
* Correspondence: sander.leeuwenburgh@radboudumc.nl
† These authors contributed equally to this work.

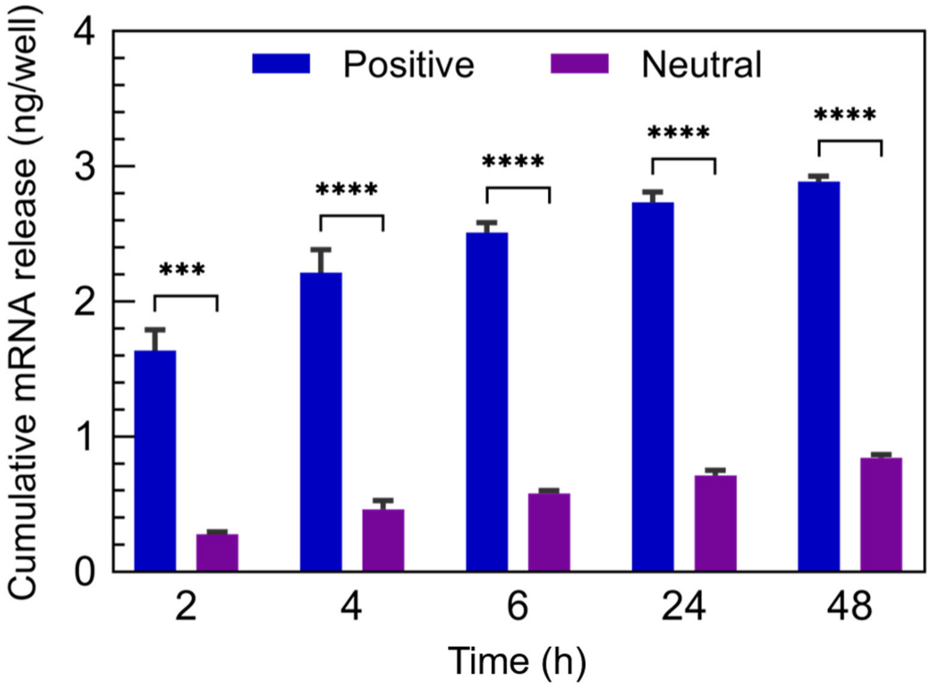

Figure S1. mRNA release from gelatin nanoparticles. Absolute amounts of mRNA released from gelatin nanoparticles over 48 h ($n = 3$). Data for negatively charged nanoparticles were not included since detected mRNA amounts were below the detection limit. Statistical significance is shown as *** $p < 0.001$ and **** $p < 0.0001$.

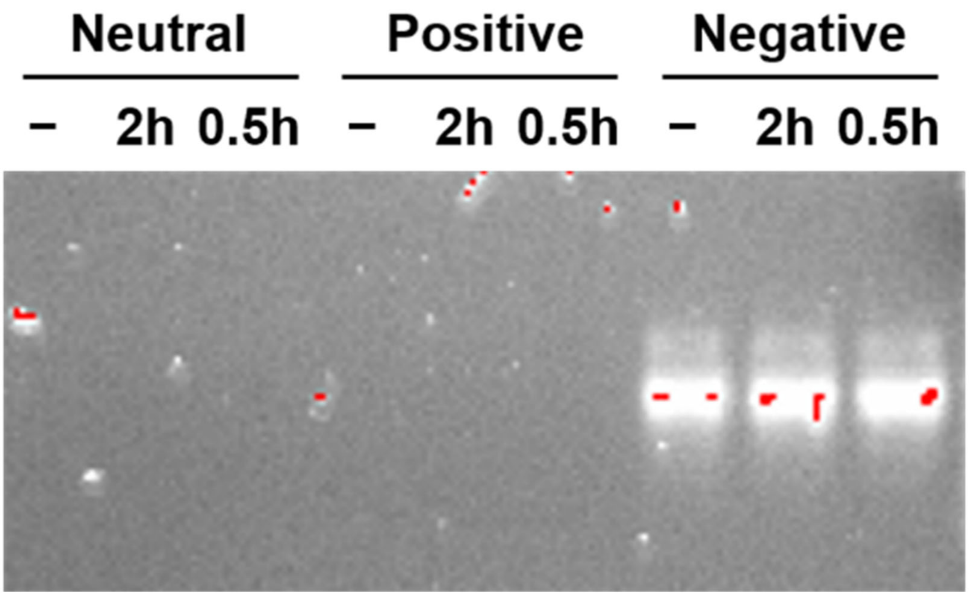

Figure S2. Loading capacity of gelatin nanoparticle. Gel electrophoresis of RNA remaining in supernatant after loading gelatin nanoparticles with RNA.

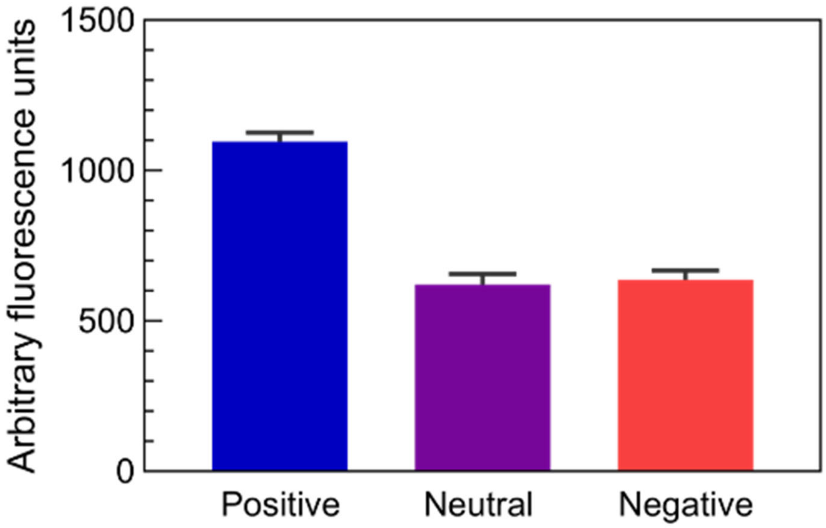

Figure S3. Fluorescent labelling efficiency of gelatin nanoparticles. Fluorescence intensity of equal amounts of fluorescently-labelled gelatin nanoparticles (n=3).

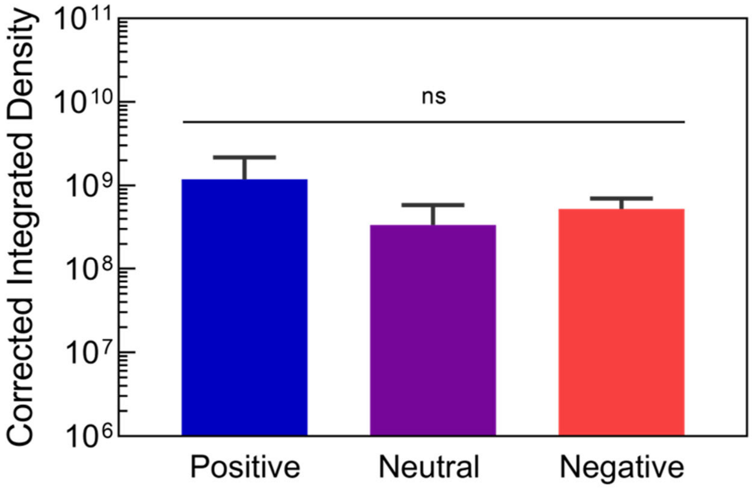

Figure S4. Internalization of gelatin nanoparticles. Quantification of internalization of gelatin nanoparticles by pre-osteoblastic cells after 24 h after normalization of original fluorescence based on Figure S3 ($n = 6$). ns indicates no statistically significant differences.

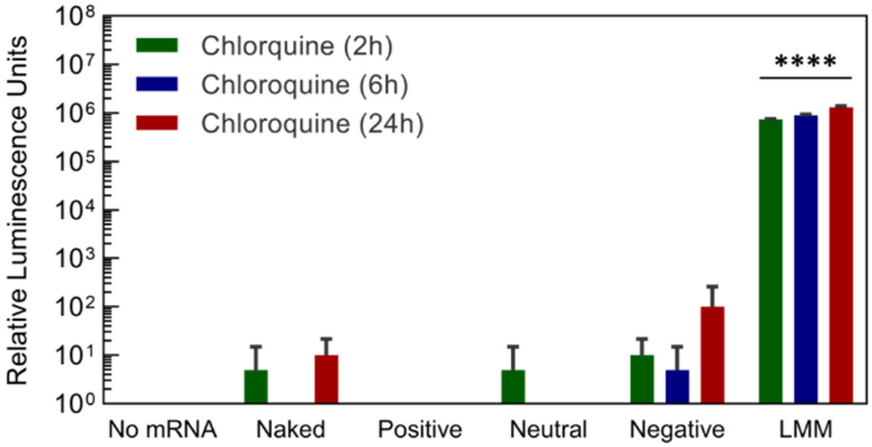

Figure S5. Endosomal release of mRNA. Expression of luciferase mRNA 50 h after transfection with differently charged gelatin nanoparticles or commercial lipoplexes (LMM) and stimulation of endosomal escape by addition of chloroquine at different time points (n=4). **** indicates statistically significant differences compared to untransfected control (no mRNA) with $p < 0.0001$.
